# Mesenchymal Stem Cell Therapy for Doxorubicin-Induced Cardiomyopathy: Potential Mechanisms, Governing Factors, and Implications of the Heart Stem Cell Debate

**DOI:** 10.3389/fphar.2019.00635

**Published:** 2019-06-14

**Authors:** Abdelrahman Ibrahim Abushouk, Amr Muhammad Abdo Salem, Anas Saad, Ahmed M. Afifi, Abdelrahman Yousry Afify, Hesham Afify, Hazem S.E. Salem, Esraa Ghanem, Mohamed M. Abdel-Daim

**Affiliations:** ^1^Faculty of Medicine, Ain Shams University, Cairo, Egypt; ^2^School of Medicine, New Giza University, Giza, Egypt; ^3^Wake Forest University, Winston-Salem, NC, United States; ^4^Faculty of Medicine, Al-Azhar University, Cairo, Egypt; ^5^Department of Pharmacology, Faculty of Veterinary Medicine, Suez Canal University, Ismailia, Egypt

**Keywords:** anthracyclines, cardiac progenitor cells, cardiomyopathy, doxorubicin, mesenchymal stem cells

## Abstract

Over the past decades, researchers have reported several mechanisms for doxorubicin (DOX)-induced cardiomyopathy, including oxidative stress, inflammation, and apoptosis. Another mechanism that has been suggested is that DOX interferes with the cell cycle and induces oxidative stress in C-kit+ cells (commonly known as cardiac progenitor cells), reducing their regenerative capacity. Cardiac regeneration through enhancing the regenerative capacity of these cells or administration of other stem cells types has been the axis of several studies over the past 20 years. Several experiments revealed that local or systemic injections with mesenchymal stem cells (MSCs) were associated with significantly improved cardiac function, ameliorated inflammatory response, and reduced myocardial fibrosis. They also showed that several factors can affect the outcome of MSC treatment for DOX cardiomyopathy, including the MSC type, dose, route, and timing of administration. However, there is growing evidence that the C-kit+ cells do not have a cardiac regenerative potential in the adult mammalian heart. Similarly, the protective mechanisms of MSCs against DOX-induced cardiomyopathy are not likely to include direct differentiation into cardiomyocytes and probably occur through paracrine secretion, antioxidant and anti-inflammatory effects. Better understanding of the involved mechanisms and the factors governing the outcomes of MSCs therapy are essential before moving to clinical application in patients with DOX-induced cardiomyopathy.

## Introduction

Anthracyclines [doxorubicin (DOX), daunorubicin, idarubicin, epirubicin, and anthraquinone] are effective chemotherapeutic agents, used in the management of several tumors. Among them, DOX is the most widely used, especially for the treatment of breast and esophageal cancers, osteosarcoma, Kaposi’s sarcoma, and Hodgkin’s and non-Hodgkin’s lymphomas (Tacar et al., 2013). Despite its efficacy, reports of DOX-induced cardiomyopathy have jeopardized its clinical use due to the high risk of developing heart failure (HF) (Chatterjee et al., 2010; Renu et al., 2018).

DOX cardiotoxicity is categorized (according to its onset) into acute or chronic. Acute cardiotoxicity usually occurs within the first 3 days of administration with an approximate incidence of 11% (Swain et al., 2003; Takemura and Fujiwara, 2007). It has a wide range of non-specific symptoms, including chest pain (due to myopericarditis) and palpitations (secondary to sinus tachycardia, paroxysmal non-sustained supraventricular tachycardia, or premature atrial and ventricular beats). Meanwhile, chronic cardiotoxicity has an incidence of 1.7% and often manifests within 30 days of drug use (Von Hoff et al., 1979). However, it may manifest after a latency period of 6 to 10 years (Ferrans et al., 1997). DOX-induced cardiomyopathy not only is dose-dependent but also is influenced by other factors, including the patient’s age, history of previous cardiovascular diseases, and reduced left ventricular ejection fraction (LVEF) (Von Hoff et al., 1979).

DOX accumulates primarily in the liver, kidneys, and the heart. The heart is highly susceptible to DOX toxicity because the mitochondria-to-cardiomyocyte ratio is elevated, making it more susceptible to oxidative stress. Further, the heart has a low regenerative capacity, compared to other organs (Barry et al., 2007). Cardiac tissues, exposed to DOX, show several structural changes, including myofibrillar loss, eosinophilic fibers, pyknotic nuclei, blood vessel congestion, and widespread infiltration of neutrophils (Abdel-Daim et al., 2017). Functionally, DOX intoxication in humans and animal models is associated with reduced fractional shortening, LVEF, reduced contractility, increased afterload, and increased isovolumetric relaxation time, all of which reflect cardiac dysfunction or even failure (Lipshultz et al., 1991; Stoddard et al., 1992).

**Figure 1:**
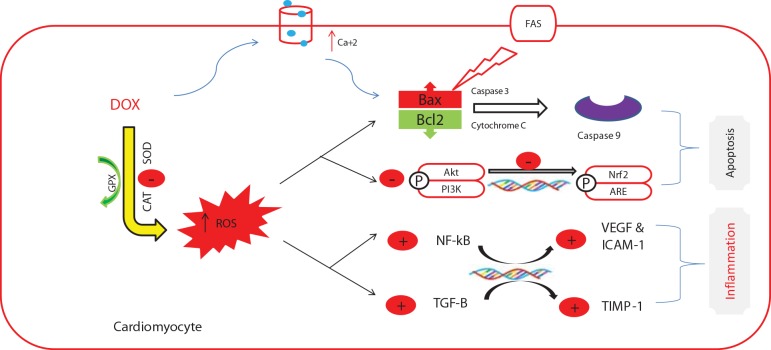
The commonly investigated mechanisms of doxorubicin-induced cardiotoxicity. This figure was adapted from a previous publication by the authors (Abushouk et al., 2017) and an adequate permission for reproduction was obtained from the publisher (Elsevier).

Several studies investigated the pathogenesis of DOX-induced cardiomyopathy and implicated various mechanisms, including oxidative stress {reactive oxygen species (ROS)-rich microenvironment], resulting in endothelial cell injury with subsequent leukocyte infiltration and cytokine secretion [interleukin (IL)-1β, IL-6, and tumor necrosis factor-α] (Pecoraro et al., 2016; Abdel-Daim et al., 2017). Further, DOX increases the expression of nuclear factor kappa-B, cyclooxygenase-2, and inducible nitric oxide synthase (Mantawy et al., 2014). These events, along with suppressed expression of bcl2 (Leung and Wang, 1999), activate the apoptotic cascade. The apoptotic cells can act as a source of damage-associated molecular patterns (DAMPs) that can be identified by the pattern recognition receptors (PRRs), exaggerating the immune response (Krysko et al., 2012). In addition, DOX downregulates the expression of cardiac-muscle-specific and mitochondrial proteins, which are essential for cardiomyocyte function (Ito et al., 1990) (**Figure 1**).

Recently, other potential mechanisms are coming into light. For example, DOX inhibits the activity of nuclear topoisomerase IIβ, which is essential for DNA replication and transcription (Lyu et al., 2007). This enzyme is abundantly present in rapidly growing cancer cells (therefore, being a target for DOX), as well as quiescent cardiac myocytes. Inhibition of the enzyme leads to DNA breaks and transcriptional alterations (Zhang et al., 2012). Further, DOX enhances the proteolytic activity of calpain enzyme through the increase of intracellular Ca levels, causing sarcomere disruption and myofibril loss (Lim et al., 2004). This plethora of potential mechanisms suggests that DOX-induced cardiotoxicity probably occurs through a multiple-mechanisms hypothesis (Mele et al., 2016).

Of all the aforementioned mechanisms, oxidative stress has received the most attention (Varricchi et al., 2018). The metabolism of DOX produces DOX–semiquinone, an unstable compound that reacts with O_2_, generating H_2_O_2_ and O_2_^•−^ (Cummings et al., 1991). DOX also inhibits mitochondrial iron export, leading to iron accumulation within the mitochondria and production of ROS (Gammella et al., 2014). These radicals cause cardiolipin peroxidation, inducing the release of mitochondrial cytochrome c enzyme, leading to more cardiolipin peroxidation (Goormaghtigh et al., 1990). In addition, DOX augments the activity of extra-mitochondrial oxidative enzymes as xanthine oxidase (Pan and Bachur, 1980) and NADPH oxidase (Zhao et al., 2010). On the other hand, DOX inhibits the activities of endogenous enzymatic and non-enzymatic antioxidants (Abdel-Daim et al., 2017; Abushouk et al., 2017). Therefore, this imbalance between ROS generation and neutralization leads to oxidative stress.

There are no specific guidelines for the amelioration or treatment of DOX-induced cardiotoxicity. The most common approach is decreasing the drug dosage. In practice, the drug is stopped if there is a 10% reduction in the LVEF from baseline (Curigliano et al., 2016). Other approaches include the administration of anthracyclines as infusions rather than as boluses or the use of liposomal DOX (Saidi and Alharethi, 2011). Dexrazoxane, an EDTA chelator, has been shown effective in ameliorating DOX-induced cardiotoxicity. However, its use is limited to patients who receive a cumulative dose of DOX > 300 mg/m^2^ (Cvetkovic and Scott, 2005). Further, beta-blockers, statins, and angiotensin antagonists might protect against systolic and diastolic dysfunction of DOX toxicity (Kalam and Marwick, 2013), but further clinical trials are required.

The impairment of cardiac repair after DOX toxicity and the occurrence of late cardiotoxic effects indicate that DOX may influence cardiac adaptation to stress or cardiac regeneration. The latter has been the axis of a major debate recently on whether regenerative stem cells exist or not in the adult mammalian heart. On one hand, some experiments have shown that DOX induces apoptosis of cardiac progenitor cells (CPCs), reducing the pool of available CPCs to regenerate the damaged, senescent, and poorly functioning myocytes (Huang et al., 2010; Spallarossa et al., 2010; Piegari et al., 2013). On the other hand, recent experiments presented strong evidence that the so-called “cardiac progenitor cells” do not have a cardiac regenerative potential in the adult mammalian heart (Van Berlo et al., 2014; Li et al., 2018).

## The Heart Stem Cell Debate

The concept of endogenous regenerative cardiac stem cells started in 2001 (Maliken and Molkentin, 2018). Quaini et al. (2002) and Orlic et al. (2001) reported that adult cardiac and bone marrow cells expressing the protein (c-kit) were able to generate new myocytes. Later reports provided further support to this finding (Beltrami et al., 2001; Beltrami et al., 2003). The notion, developed based on these results, was that under normal circumstances, minor cardiac damage causes progenitor cells to migrate to the site of injury and differentiate into cardiac cells (Chimenti et al., 2003; Huang et al., 2010). Therefore, the decline of the progenitor cell pool, cellular senescence, and loss of their functions (as claimed to occur after DOX exposure) lead to impaired cardiac homeostasis and inability of the heart to cope with continuous stress (Chimenti et al., 2003; Spallarossa et al., 2010; Cesselli et al., 2011). Other authors suggested that these cells can be used in cell-based therapy after they are withdrawn from cardiac biopsies and expanded ex vivo (Messina et al., 2004). C-kit+ cells were evaluated for their regenerative capacity in animal models with myocardial infarction (MI) (Barile et al., 2007), as well as in humans (Bolli et al., 2011; Makkar et al., 2012; Dixit and Katare, 2015).

The debate started in 2004 when three investigations by Murry et al. (2004), Balsam et al. (2004), and Nygren et al. (2004) concluded that C-kit+ hematopoietic stem cells could not differentiate into cardiomyocytes, even in the microenvironment of the injured heart. Another paper by Van Berlo et al. (2014) in 2014 confirmed these conclusions that C-kit+ cannot differentiate into cardiac myocytes. However, the cell lineage tracing technique of the latter paper was criticized (Vicinanza et al., 2018). A year later, another research group reported that C-kit+ cells can differentiate into cardiac myocytes *in vitro* when the optimal conditions are provided. The authors argued that the cells have the potential; however, the microenvironment in the heart after MI does not allow them to perform such differentiation (Hatzistergos et al., 2015). A recent study in 2018 used a dual genetic lineage tracing system and showed that non-myocytes could generate myocytes in the embryonic stage, but not in the adult homeostatic condition or after MI (Li et al., 2018). Later in the same year, several basic science studies were retracted or followed with an editorial expression of concern due to evidence of data falsification or image manipulation. Concurrently, the National Heart, Lung, and Blood Institute (NHLBI) stopped its ongoing CONCERT-HF study, which was testing the regenerative efficacy of the combination of MSCs and C-kit+ cells in patients with HF due to safety concerns.

## The Effects of DOX on C-kit+ Cells With Regard to the Recent Debate

The main debate is whether C-kit+ cells can give origin to new myocytes and there is growing evidence that they cannot; however, they may be involved in cardiac repair through other mechanisms. Therefore, impairment of their functions upon DOX exposure might contribute to the observed late toxic effects of DOX. Below is a brief review of the published reports on the effects of DOX on C-kit+ cells that should be revisited in light of the piling evidence, doubting their regenerative capacity.

Huang and colleagues conducted an experiment on a juvenile mouse model to study the mechanism of late-onset DOX cardiomyopathy. They found that treatment with DOX caused a permanent decline in the number of C-kit+ and endothelial progenitor cells (EPCs) in treated mice hearts, as well as telomeric shortening and progressive cell senescence. Moreover, DOX-treated mice became more susceptible to ischemic injuries and MI, and less capable of responding even to minor stresses (Huang et al., 2010). Other studies were conducted on isolated human C-kit+ cells, EPCs, and living rats. DOX-treated cells showed reduced viability and increased apoptosis. After a 6-week period, the myocardium showed almost complete depletion of these cells (Spallarossa et al., 2010; De Angelis et al., 2010). Researchers in another experiment isolated C-kit+ cells from the hearts of DOX-treated patients who died due to cardiomyopathy or other reasons (the primary disease for example) and compared them to C-kit+ cells, isolated from autopsies of patients, not treated with DOX. They found significantly higher cellular senescence in cells obtained from DOX-treated patients. When control cells were treated with DOX, similar effects occurred. To study the persistence of DOX effects on C-kit+ cells, the authors washed the cells from DOX and left them to grow and compared the results with those obtained early after exposure. After a week, the cells showed markedly less apoptosis and higher vitality. However, they still expressed higher senescence, which indicates the long-term toxic effects of DOX (Piegari et al., 2013).

Several mechanisms were suggested to explain the above findings. For example, DOX alters the molecular regulators of the cell cycle, causing cell cycle arrest. The activity of telomerase is also important for the proliferation of progenitor cells. DOX was shown to decrease the activity of telomerase, causing senescence of C-kit+ cells (Huang et al., 2010). Another possible mechanism is the generation of ROS (Spallarossa et al., 2010), which cause damage to myocytes (Doroshow, 1983; Takemura and Fujiwara, 2007). This was proven *in vivo* and *in vitro* as anthracyclines were found able to promote oxidative stress in isolated human C-kit+ cells and in living mice (De Angelis et al., 2010; Spallarossa et al., 2010; Piegari et al., 2013). This DNA damage caused by oxidative stress later leads to over- or underexpression of molecular regulators of cell cycle (mainly P53 and Rb genes) (Piegari et al., 2013), which, in turn, causes apoptosis or cellular senescence (Levine, 1997; De Angelis et al., 2010).

In addition, DOX increases the expression of P16^INK4A^ (a marker of cellular senescence that causes cell cycle arrest in the G1 phase) in progenitor cells (Spallarossa et al., 2010; Piegari et al., 2013) through JNK or P38 activation (Wada et al., 2008; Spallarossa et al., 2010). Cyclin D1 and cyclin-dependent kinase 4 (CDK-4) are molecules that work together to phosphorylate the Rb protein into phospho-Rb^Ser798^ protein that stops the inhibition of the cell cycle progression (Piegari et al., 2013). Isolated C-kit+ cells after DOX treatment showed a substantial decrease in the levels of cyclin D1, CDK-4, and/or phospho-Rb^Ser798^ (De Angelis et al., 2010). Growth factor receptors like insulin-like growth factor-1 (IGF-1) receptor and hepatocyte growth factor receptors (c-Met) are also expressed by C-kit+ cells, and stimulation of these receptors is thought to be crucial for myocyte regeneration in response to injury (D’amario et al., 2011; Piegari et al., 2013). DOX-exposed C-kit+ cells showed reduced expression of IGF-1 receptors and c-Met (Piegari et al., 2013). Therefore, impaired growth factors functions may be another possible mechanism of DOX-induced cardiomyopathy.

The recent findings negating the regenerative capacity of the C-kit+ cells do not necessarily contradict the published observations about DOX effects on these cells. DOX may reduce the ability of these cells to engage in other cardiac repair activities rather than cardiac regeneration. Further research is needed to characterize the structural and functional aspects of C-kit+ cells.

## Stem Cell Therapy for DOX-Induced Cardiomyopathy

Stem cell therapy is a promising modality for DOX-induced cardiomyopathy; however, it is still in the preclinical stage. Several studies investigated the role of regenerative medicine as an alternative therapy to heart transplantation (Dimmeler et al., 2005; Pelacho et al., 2007; Mathur et al., 2017). In particular, mesenchymal stem cells (MSCs) have many properties that make them a suitable choice for the prevention and treatment of myocardial diseases, including DOX-induced cardiac damage.

First, MSCs secrete paracrine factors that are involved in the cardiac remodeling process (Garbade et al., 2009). These factors have different functions. For example, IGF-1 increases cell proliferation and inhibits apoptosis by improving the mitochondrial function in different cell types including cardiomyocytes (Duerr et al., 1995; Lai et al., 2003). Some factors have angiogenic functions, e.g., endothelin-1, platelet-derived growth factor, and vascular endothelial growth factor, or participate in the regulation of sympatho-adrenal axis and normalization of noradrenaline and adrenaline plasma levels (Dhein et al., 2006). To confirm the value of paracrine secretion in MSC-observed beneficial effects, Zhang et al. (2015) reported that only the conditioned medium of bone marrow MSCs (BMMSCs) and induced pluripotent stem cells (iPSCs) can alleviate DOX-induced HF, cell apoptosis, and cardiac fibrosis.

Moreover, MSCs ameliorate oxidative stress by controlling redox microenvironment (increasing heme-oxygenase-1 expression and reducing 8-OHdG tissue concentration) and consequently inhibiting apoptosis induced by ROS (Liu et al., 2012; Jin et al., 2013; Calió et al., 2014). Other investigators reported that MSCs have high resistance to oxidative stress, high concentrations of glutathione, and high basal gene expression of glutathione peroxidase, superoxide dismutase, and catalase (Valle-Prieto and Conget, 2010). Xia and Hou (2018a) showed that MSC treatment significantly ameliorated DOX-induced senescence in H9c2 cells as marked by the reduced expression of P53 and P16, probably by inhibiting the miR34aSIRT1 axis.

**Figure 2:**
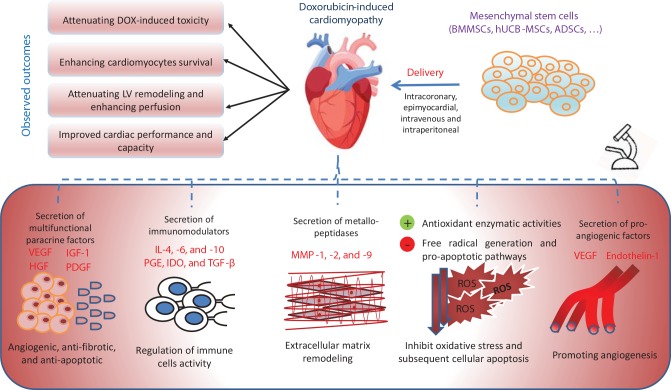
The mechanisms of action of stem-cell-based treatment in ameliorating doxorubicin-induced cardiotoxicity. ADSCs, adipose-derived stem cells; BMMSCs, bone-marrow mesenchymal stem cells; HGF, hepatocyte growth factor; hUCB-MSCs, human umbilical cord blood mesenchymal stem cells; IDO, indoleamine 2,3-dioxygenase; IGF-1, insulin-like growth factor-1; Il, interleukin; MMP, matrix metallopeptidase; PDGF, platelet-derived growth factor; PGE, prostaglandin E; ROS, reactive oxygen species; TGF-β, transforming growth factor-β; VEGF, vascular endothelial growth factor.

They also exert anti-inflammatory effects by modulating the functions of different immune cells; they can activate, suppress, and control migration and differentiation of T and B lymphocytes, macrophages, natural killer cells, dendritic cells, and neutrophils by the secretion of different immunomodulators, e.g., IL-4, IL-6, IL-10, prostaglandin E2, transforming growth factor-β, and indoleamine 2,3-dioxygenase. Further, MSCs express Toll-like receptors, e.g., TLR3 and TLR4, which play an important role in the inflammatory process (Liotta et al., 2008). In addition, MSCs have been shown to perform an anti-fibrotic function by secreting matrix metallopeptidase-9 (Ankrum and Karp, 2010; Samper et al., 2013) (**Figure 2**).

Although MSCs can express some cardiomyocyte markers (Kawada et al., 2004; Gopinath et al., 2010), their settlement in the myocardium was weak even with the local route of administration. Therefore, myocardial regeneration does not involve differentiation of MSCs into cardiomyocytes (Kang et al., 2012). Now, the overall consensus is that C-kit+ cells do not differentiate into cardiomyocytes, and it is not likely that any other stem cell is more cardiogenic (Maliken and Molkentin, 2018).

In addition to the reported beneficial effect of systematic administration of MSCs on DOX-induced cardiomyopathy, they showed significant protection for other organs as well. They were found to decrease DOX-induced nephropathy by decreasing glomerular inflammation and sclerosis (Zoja et al., 2012). Moreover, the anti-inflammatory effect of MSCs makes them protective against DOX-induced inflammation in the brain (Jansen et al., 2008) and the liver (Tulubas et al., 2015).

## Factors That Influence the Outcome of MSC Therapy for DOX Cardiomyopathy

The varying results of published experiments on the benefit of MSC use in models of DOX-induced cardiomyopathy indicate that there are several factors that affect the outcome of this treatment. Below is a summary of the most investigated factors in this regard (**Table 1**).

**Table 1:** Summary of the methods and experimental findings on using mesenchymal stem cell therapy for DOX-induced cardiotoxicity.

Study ID	Animal model	Doxorubicin dose and route	MSCs type and source	MSCs dose	Route and time of MSC administration	Findings
Chen et al. (2006)	Rabbit	2 mg/kg per week for 8 weeks (i.p.)	BMMSCs or skeletal myoblasts (autologous)	1 × 10^7^	Intracoronary, 4 weeks after Dox treatment	The LVEF was not significantly improved in either the BMMSCs or skeletal myoblast-treated groups.
Garbade et al. (2009)	Rabbit	3 mg/kg for 6 weeks (i.p.)	BMMSCs (autologous)	1.5/2 × 10^6^	Epimyocardial, 2 weeks after Dox treatment	BMMSCs treatment significantly increased the LVEF. On histological examination, cell-treated hearts exhibited less collagen content and higher capillary density. However, the transplanted cells did not show any cardiac markers.
Mohammadi Gorji et al. (2012)	Rat	Three doses of 2.5 mg/kg per week for 2 weeks (i.p.)	BMMSCs (heterologous)	5 × 10^6^	Intravenous, 2 weeks after Dox treatment	Both MSCs and its conditioned medium significantly reduced myocardial fibrosis and Bcl-2 expression. Compared to the standard medium, the MSC-conditioned medium had significantly higher levels of HGF and IGF.
Yu et al. (2014)	Rat	2.5 mg/kg per week for 6 weeks (i.p.)	BMMSCs (heterologous)	5 × 10^6^	Intravenous, one injection per day (10 times)/10 weeks after Dox treatment	The survival rate and LVEF in rats treated with MSCs, compared to placebo-treated rats. Further, MSC treatment reduced myocardial collagen volume fraction and mRNA expression of TGF-β1, AT1, and CYP11B2.
Ammar et al. (2015)	Rat (diabetic)	2.5 mg/kg 3 times/week for 2 weeks (i.p.)	BMMSCs and ADSCs (from human tissues)	2 × 10^6^ for either cell type	Intravenous, 4 weeks after the last DOX injection	BM-MSCs and ADSCs were equally effective in alleviating DOX-induced cardiac damage by decreasing immune cell infiltration and collagen deposition and enhancing angiogenesis
Dias et al. (2015)	Rat	3.75 mg/kg/day once a week for 4 weeks (i.p.)	BMMSCs and skeletal myoblasts (autologous)	5 × 10^4^	Subepicardial, 4 weeks after the last dose of DOX	The combined stem cell treatment significantly improved the LVEF, compared to the saline-treated group. Histological examination showed proliferation of skeletal muscle cells in the myocardium.
Zhang et al. (2015)	Mouse	3 mg/kg, 3 times per week for 2 weeks (i.p.)	Conditioned medium from BMMSCs (BMMSCs-CdM) or iPSC-derived MSCs	50 μl of MSCs-CdM	Intramyocardial, after creation of the DOX-induced cardiomyopathy model	Compared to BM-MSCs-CdM, iPSC-MSCs-CdM treatment exhibited better alleviation of heart failure, as well as less cardiomyocyte apoptosis and fibrosis.
Deng et al. (2017)	Rat	2.5 mg/kg for 2 weeks (i.p.)	BMMSCs (cells with or without Nkx2.5 transfection)	1 × 10^7^	Intravenous, 3 weeks after Dox treatment	The LVEF was increased by 43.4% and 49.9% in rats treated with BMMSCs and Nkx2.5-transfected BMMSCs, respectively. Further, Nkx2.5 transfection improved MSCs differentiation into cardiomyocyte-like cells and reduced myocardial fibrosis.
Zeng et al. (2017)	Rat	2 mg/kg seven times in 2 days (i.p.)	BMMSCs (heterologous) with and without miR-21 over expression		i.p., injection after cardiotoxicity induction	BMMSCs, overexpressing miR-21, exhibited more proliferation than untransfected cells and significantly enhanced expression of Bcl-2, VEGF and Cx43 and reduced expression of Bax, BNP and troponin T
Soliman et al. (2017)	Rat	450 mg/m^2^ for 3 consecutive days (i.p.)	BMMSCs (heterologous) with and without sodium valproate and electric stimulation (ES) over the shoulder	5 × 10^5^	Intravenous, after DOX cardiotoxicity induction	Rats treated by BMMSCs and valproate/ES combination showed similar biochemical parameters to the control group, as well as better histopathological appearance and cardiac homing of MSCs than rats treated by stem cells alone.
Oliveira et al. (2013)	Rat	5 mg/kg weekly for 4 weeks (i.p.)	ADSCs (heterologous)	3 × 10^6^	Intravenous, prior to the beginning of the experiment	Unlike *C. sinensis* extract, treatment by ADSCs significantly improved the LVEF. However, no cell engraftment was detected in the host cardiac tissue.
Pınarlı et al. (2013)	Rat	12 mg/kg as a single dose (i.p.)	ADSCs (alone or with resveratrol)	2 × 10^6^	i.p., starting the day after DOX injection, then two times at 5 days interval	The best hemodynamic (left ventricular end diastolic pressure and the rate of pressure development, yet not significant) and histological outcomes were observed in the group, treated by resveratrol and ADSCs.
Gopinath et al. (2010)	Mouse and cultured neonatal rat cardiomyocytes	400 ng/kg per minute (oral)	hUCB-MSCs (human placenta)	2.5 × 10^6^	Intravenous, 2 weeks after Dox treatment	hUCB-MSCs exhibited differentiation into cardiomyocyte-like cells, reversed the pathological effects of DOX on cultured myocytes, and induced a shift from pathological hypertrophy towards physiological hypertrophy
Di et al. (2012)	Mouse	Three cycles of 3 doses of 2 mg/kg per week (i.p.)	hUCB-MSCs (human placenta)	1 × 10^6^	Intravenous, at the end of each Dox cycle	MSC treatment significantly reduced myocardial necrosis and increased LVEF and fractional shortening, probably through reduction of oxidative stress. Further, MSCs treatment had no effect on tumor growth.
Mousa et al. (2018)	Rat	1.25 mg/kg every other day for 1 month (i.p.)	hUCB-MSCs (human placenta) with carvedilol	1.5 × 10^6^	Intravenous, single dose, along with carvedilol administration	The combination of hUCB and carvedilol reduced DOX-induced electrocardiographic abnormalities and cardiac concentrations of oxidative stress markers and caspase-3, while increased cardiac concentrations of VEGF and IGF-1
Abd Allah and Hussein (2017)	Rat	2.5 mg/kg every other day for 2 weeks (i.p.)	hUCB-MSCs (human placenta)	5 × 10^6^	Intravenous, 1 week after the last DOX dose	Treatment by hUCB-MSCs resulted in significant amelioration of DOX-induced oxidative stress, ECG abnormalities and histopathological alterations.
Mao et al. (2017)	Rat	2 mg/kg once a week for 8 weeks (i.p.)	hUCB-MSCs (human placenta)	2.5 × 1 0^5^ (low dose group) and 1 × 10^6^ (high dose group)	Intramuscular, 2 weeks after the last DOX injection	Treatment by hUCB-MSCs significantly increased LVEF, as well as the expression of VEGF, IGF-1, and HGF in the myocardium and attenuated mitochondrial swelling and maintained sarcolemma integrity
Haydardedeoglu et al. (2018)	Rat	2.5 mg/kg on day 1 and 4 mg/kg on day 21	Fetal-derived MSCs	2 × 10^6^	i.p., on days 7, 14, and 21 after the last DOX injection	The intraperitoneal route can be a valid alternative to the intravenous and intra-cardiac routes.

ADSCs, adipose-derived stem cells; AT-1, angiotensin 1 receptor; BMMSCs, bone-marrow derived mesenchymal stem cells; BNP, brain naturetic peptide; hUCB, human umbilical cord blood; IGF, insulin-like growth factor; LVEF, left-ventricular ejection fraction; TGF-β1, transforming growth factor-β; VEGF, vascular endothelial growth factor.

**(A) MSC type:** Two main types of stem cells are present: embryonic stem cells (ESCs), present in the inner cell mass of the blastocyst, and adult stem cells, present in different mature tissues to replace dead cells (Swain et al., 2003; Takemura and Fujiwara, 2007). Embryonic stem cells have two considerations that hinder their use; first is the ethical consideration regarding destruction of a developing embryo, and second is the possibility of rejection and the need for lifelong immunosuppressive drugs to maintain the graft (Pfister et al., 2014). Further, the risk of rejection is still present due to potential incompatibility. iPSCs are adult cells, genetically reprogrammed to express genes and factors, required to maintain the properties of ESCs (Choi et al., 2009). Therefore, they overcome the ethical and the rejection problems because they can be withdrawn from the patient (autologous graft). On the other hand, MSCs are able to enhance C-kit+ cell proliferation and differentiation, which may ameliorate the DOX toxicity on the long run (Hatzistergos et al., 2010; Loffredo et al., 2011). The bone marrow, adipose tissue, and umbilical cord blood are the most common sites for MSC extraction. The most commonly used cells in the reviewed studies were BMMSCs (Chen et al., 2006; Garbade et al., 2009; Mohammadi Gorji et al., 2012; Yu et al., 2014; Ammar et al., 2015; Dias et al., 2015; Deng et al., 2017; Soliman et al., 2017; Zeng et al., 2017) and human umbilical cord blood (hUCB)-MSCs (Gopinath et al., 2010; Di et al., 2012; Abd Allah and Hussein, 2017; Mao et al., 2017; Mousa et al., 2018). Fewer studies used adipose-derived stem cells (ADSCs) (Oliveira et al., 2013; Pınarlı et al., 2013) and fetal-derived stem cells (Haydardedeoglu et al., 2018). The majority of these studies showed promising effects; however, to make a conclusion about the most optimal type, head-to-head comparisons are required.

**(B) Dose of injected MSCs:** In the published studies, the number of injected MSCs in DOX-intoxicated animal models ranged from 5 × 10^4^ to 1 × 10^7^ cells. Chen et al. (2006) used the highest number of BMMSCs (1 × 10^7^ cells), and the results were not satisfactory as regards LVEF. They found further deterioration as regards abnormalities in local ventricular conduction in DOX-treated hearts, as well as positive correlation between the dispersion of the activation time and the number of cells derived from stem cells in the pacing site. Another study used a similar dose and reported beneficial effects, especially when BMMSCs were transfected with Nkx2.5 (Deng et al., 2017). Mao et al. (2017) used two different doses (2.5 × 10^5^ versus 1 × 10^6^) of hUCB-MSCs and reported no dose–effect relationship on LVEF.

**(C) Route of administration of MSCs:** Two routes of administration are reported in the literature: the local route [either *via* intracoronary injection by using two balloons introduced through the left common carotid artery (Chen et al., 2006) or *via* epimyocardial injection (Garbade et al., 2009)] and the systemic route by intravenous injection of MSCs (Di et al., 2012; Yu et al., 2014). The latter was the most commonly used in the reviewed studies, while few studies used the intraperitoneal (Pınarlı et al., 2013; Haydardedeoglu et al., 2018) or the intramuscular routes (Mao et al., 2017), reporting their safety as possible alternatives.

Chen and colleagues have found that the LVEF was not significantly improved by intracoronary administration of BMMSCs after DOX treatment (Chen et al., 2006). However, Garbade et al. (2009) reported that epimyocardial administration of BMMSCs, after DOX treatment, resulted in significant improvement of the LVEF. Similarly, Yu et al. (2014) concluded that systemic administration of BMMSCs resulted in significant reduction of myocardial fibrosis, as well as significant improvement of LVEF. Further, Di et al. (2012) reported significant improvement of the LVEF after systemic administration of hUCB at the end of each DOX cycle.

**(D) Time of administration:** The published studies used two different timings for administering MSCs. The first is a preventive strategy that entails administering stem cells before, during, or just after DOX administration (Di et al., 2012). The second involves administering stem cells after cardiomyopathy has been established (regenerative strategy) (Gopinath et al., 2010). Di et al. (2012) have adopted the preventive strategy (by systemic administration of hUCB at the end of each DOX cycle) that resulted in both histological (reduced apoptosis) and functional improvements (significant increase in LVEF), while Gopinath et al. (2010) have adopted the regenerative strategy (by systemic administration of hUCB-MSCs, 2 weeks after DOX treatment) that resulted in histological improvement in the form of decreased fibrosis (2.9% compared to 7.2% when mice were treated by DOX alone); however, the functional status was not investigated.

Further, studies that adopted the regenerative strategy used different intervals between DOX administration and MSC administration. Early epimyocardial administration of BMMSCs after 2 weeks of DOX treatment has resulted in significant improvement of LVEF (Garbade et al., 2009), while insignificant improvement was noted following intracoronary administration of BMMSCs, 4 weeks after DOX injection (Chen et al., 2006).

**(E) Degree of DOX-induced cardiac injury:** The improvement in cardiac function after MSC treatment can be related to the degree of cardiac affection by DOX and the pretreatment functional status. Administration of a total dose of 16 mg/kg of DOX in a rabbit model has resulted in a pretreatment EF of 77.6 ± 1.6% in the MSC group. Comparing this EF to the control group 81.5 ± 1.5% shows that there was no marked reduction in EF after DOX treatment, and this led to nonsignificant improvement in LVEF (Chen et al., 2006), while administration of a total dose of 18 mg/kg of DOX in a rabbit model resulted in a pretreatment EF of 33 ± 1.3% in the MSC group. Comparing this EF to the control group (around 41%) shows a significant reduction in EF after DOX treatment, which was improved later by stem cell treatment (Garbade et al., 2009). Another study (Yu et al., 2014) may support this conclusion; in a rat model, DOX treatment has resulted in a pretreatment EF of 66 ± 1.4% in the MSC group, compared to 88.9 ± 0.8% in the control group. These data indicate that minimal functional impairment of the heart after DOX administration results in nonsignificant improvement after MSC treatment. However, more studies are needed to verify this conclusion.

**(F) Time of assessment:** The time of assessment and follow-up duration may impact the results of MSC treatment of DOX cardiotoxicity, depending on the time given to the stem cells to exert their effects. Chen et al. (2006) have related the nonsignificant improvement in LVEF partly to the number of animals and the short follow-up time, which may have underestimated the improvement of LVEF after stem cell infusion.

**(G) Addition of supporting elements:** Some studies investigated whether editing the genetic properties of MSCs or supplementing them with pharmacological compounds can enhance their therapeutic efficacy. For example, Deng et al. (2017) reported that Nkx2.5 transfection improved MSC differentiation into cardiomyocyte-like cells (as claimed by the authors) and reduced myocardial fibrosis. Nkx2.5 is a gene that encodes a homeobox-containing transcription factor that is essential for proper heart formation and development (Jamali et al., 2001). Another study by Zeng et al. evaluated the effect of miR-21 overexpression in BMMSCs. They reported that BMMSCs, overexpressing miR-21, exhibited more proliferation than untransfected cells and significantly enhanced expression of Bcl-2, VEGF, and Cx43 and reduced expression of Bax and troponin T (Zeng et al., 2017).

Other studies explored the value of pharmacological support for MSCs function. Mousa et al. reported that the combination of hUCB-MSCs and carvedilol reduced DOX-induced electrocardiographic abnormalities and cardiac concentrations of oxidative stress markers and caspase-3. This may be due to its antioxidant and anti-inflammatory effects (Mousa et al., 2018). DOX has been reported to induce oxidative stress and senescence in MSCs (Xia and Hou, 2018b; Kozhukharova et al., 2018). Therefore, supplementation with antioxidants like resveratrol may be a promising strategy for future studies (Pınarlı et al., 2013; Shaban et al., 2017). In another study, the cardiac tissues of rats treated by BMMSCs and sodium valproate combination showed better histopathological appearance and cardiac homing of MSCs than rats treated by stem cells alone. To enhance homing of BMMSCs, Soliman et al. investigated whether supplementing BMMSCs with valproate along with an electric current over the shoulder would enhance cardiac homing. They concluded that this pharmaco-electrical method can be beneficial in enhancing MSCs homing in the injured myocardium (Soliman et al., 2017).

All the previous factors may affect the outcomes of stem cell treatment. More studies are needed to investigate and fulfill all these factors to know the optimal cell type, dose, and route of administration, as well as the proper time interval between their use and DOX injection.

## Discussion and Implications for Future Research

The experiments reviewed here demonstrated favorable effects for administering MSCs in DOX-induced cardiomyopathy through diverse mechanisms. They also showed that the efficacy of MSC therapy for this condition depends on several factors that must be further studied and accommodated for. Some lessons for further preclinical experiments and future trials can be learnt from discussing the previous trials on MSC therapy for other cardiovascular conditions as HF and ischemic cardiomyopathy.

Stem cell-based therapies have been tested in numerous HF and MI trials and were proven to be clinically feasible (Sanganalmath and Bolli, 2013). However, it has recently become evident that the improvements observed in these trials are not because of direct differentiation of stem cells into cardiomyocytes, but because of the immune regulation, angiogenic abilities, and paracrine factors produced by these cells (Eschenhagen et al., 2017). The extent of cardiac performance improvement in these trials varies as well. Some trials in ischemic cardiomyopathy reported significant satisfactory improvements, while others reported significant yet modest improvement (Fisher et al., 2015). The heterogeneity of their results can be attributed to the differences in the used stem cell type, the routes of administration, and timing of the interventions. Despite these limitations, clinical trials are continuing, while a more comprehensive understanding of the basic science is still required.

Although several studies have been conducted to test the effectiveness of stem cell therapy in human subjects, the optimal conditions for the therapy are still debated. Regarding the timing of administration, delivery of the stem cells within the first week of insult seems to be more beneficial. Further, we would expect that higher doses of cells would be associated with more significant results. However, as indicated before, this is not necessarily the case (Chen et al., 2006; Mao et al., 2017). With regard to the cell type, various stem cell types have demonstrated the ability to improve cardiac functions; however, certain types are generally less recommended. For example, authors using skeletal myoblasts have reported ventricular arrhythmias in their subjects, suggesting lack of synchrony of these cells (Sanganalmath and Bolli, 2013). Further, it has been shown that MSCs may increase the resistance of tumor cells to DOX (Chen et al., 2014). However, other studies have revealed contradictory findings; that is, MSCs increase tumor chemosensitivity to DOX (Klopp et al., 2011; Kucerova et al., 2013). Another study on mice and humans with metastasis hinted that some MSC populations may aggravate tumor growth, suggesting a tumorigenic potential for MSCs (Kudo-Saito, 2015). However, a meta-analysis of 36 clinical studies found no association between MSC treatment and tumor development (Lalu et al., 2012). Further research and long-term follow-up are needed to establish the safety of MSC therapy.

Despite these limitations and uncertainties, cell-based therapy remains a viable option for the treatment of DOX-induced cardiotoxicity because the subjects in these trials were patients who suffered from severe cardiac injury (from ischemia) and have markedly decreased LVEF. On the contrary, patients treated with DOX are less likely to be as severely injured. Additionally, the mechanisms of cardiac insult in the two conditions are different. Whether cell therapy is going to be a clinical solution for DOX cardiomyopathy and other cardiac conditions is a question that will be answered with time and further investigations. For now, this potential therapeutic approach needs more time in the bench before moving to the clinic.

Overall, the reviewed studies reported favorable effects for administering MSCs in DOX-induced cardiomyopathy through diverse mechanisms; however, these mechanisms are unlikely to involve cardiogenic differentiation. These effects may depend on several factors, including the cell type, dose, and route of administration. Better understanding of the involved mechanisms and the factors governing the outcomes of MSC therapy is essential before moving to clinical application in patients with DOX-induced cardiomyopathy.

## Author Contributions

AIA contributed to idea conception and designing the manuscript structure. AAS, AS, and AMA contributed to searching the literature and summarizing the published reports on the topic. All authors contributed to writing and revising the manuscript draft and approved it for publication.

## Conflict of Interest Statement

The authors declare that the research was conducted in the absence of any commercial or financial relationships that could be construed as a potential conflict of interest.

## Abbreviations

BMMSCs, bone-marrow mesenchymal stem cells; DOX, doxorubicin; EPCs, endothelial progenitor cells; hUCB, human umbilical cord blood; iPSCs, induced pluripotent stem cells; MSCs, mesenchymal stem cells.
